# 

**Published:** 1916-09-02

**Authors:** 


### CONTENTS.

| | PAGE |
|---|---|
| **The Medical School's Entry in War Time** | 499 |
| **Woman's Work in Medicine After the War** | 500 |
| **Hospital and Institutional News** | 501 |
| The Hospital's Educational Number—The late Sir R. B. Martin and the Sunday Fund—Sudden Death of a Hospital Chairman—The Results of Wound Treatment in Military Hospitals—A Point for the Cambridge Medical School—Anthrax in the Shaving-brush—East Africa and the Red Cross—The New Hospital on the Mersey—A Special Hospital for Canadians—"Housed Worse than Cattle"—Disabled Officers at Regent's Park—A "Uniform System" in India—Large Bequests by the Chairman of the Bon Marché—An Institutional Abuse Ventilated in the Daily Press—War Conditions in Scottish Asylums—The Anti-Tuberculosis Campaign in Aberdeen—A New Subject for War Hospital Gazettes—This Week's Drug Market. | |
| **Research in Medicine** | 505 |
| Its Need of Endowment and Outlook. | |
| **The Royal Naval Medical Service** | 506 |
| Its Claims and Advantages. Qualifications and Regulations. | |
| **Compulsory Service in the R.A.M.C.** | 507 |
| How the Profession has Avoided Direct Governmental Control. | |
| **The Royal Army Medical Corps** | 508 |
| How Matters Stand To-day. | |
| **The Public Health Service** | 512 |
| Its Wide Field, Needs and Future. | |
| **A Medical Classic** | 513 |
| Sir James Mackenzie on Heart Affections. | |
| **Dentistry as a Profession** | 515 |
| Its Drawbacks and Preparations to Qualify. | |
| **The British Hospitals Association** | 516 |
| **The Medical Curriculum** | 517 |
| **The Medical Student and his Work** | 520 |
| The Medical Schools of the United Kingdom. | |
| **The Public Services** | 526 |
| Royal Naval Medical Service. Royal Army Medical Corps. Indian Medical Service. Colonial Medical Service. | |
| **London Post-Graduate Institutions** | 527 |
| **Tropical Medicine** | 527 |
| **Graduate Study in London Special Hospitals** | 528 |
| **The Institutional Library** | 530 |
| **"A Jumbled Argument"** | 531 |
| **The Editor's Letter-Box** | 531 |
| The Future of Insurance Practice. | |

